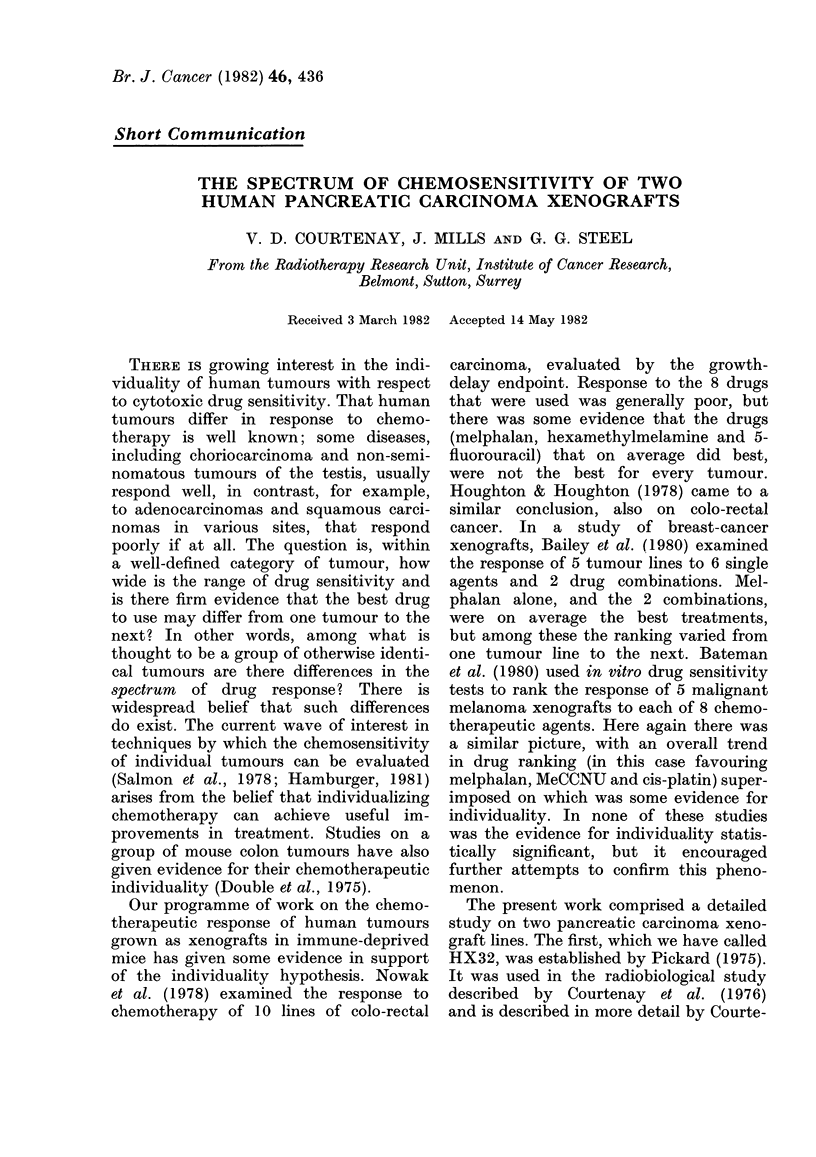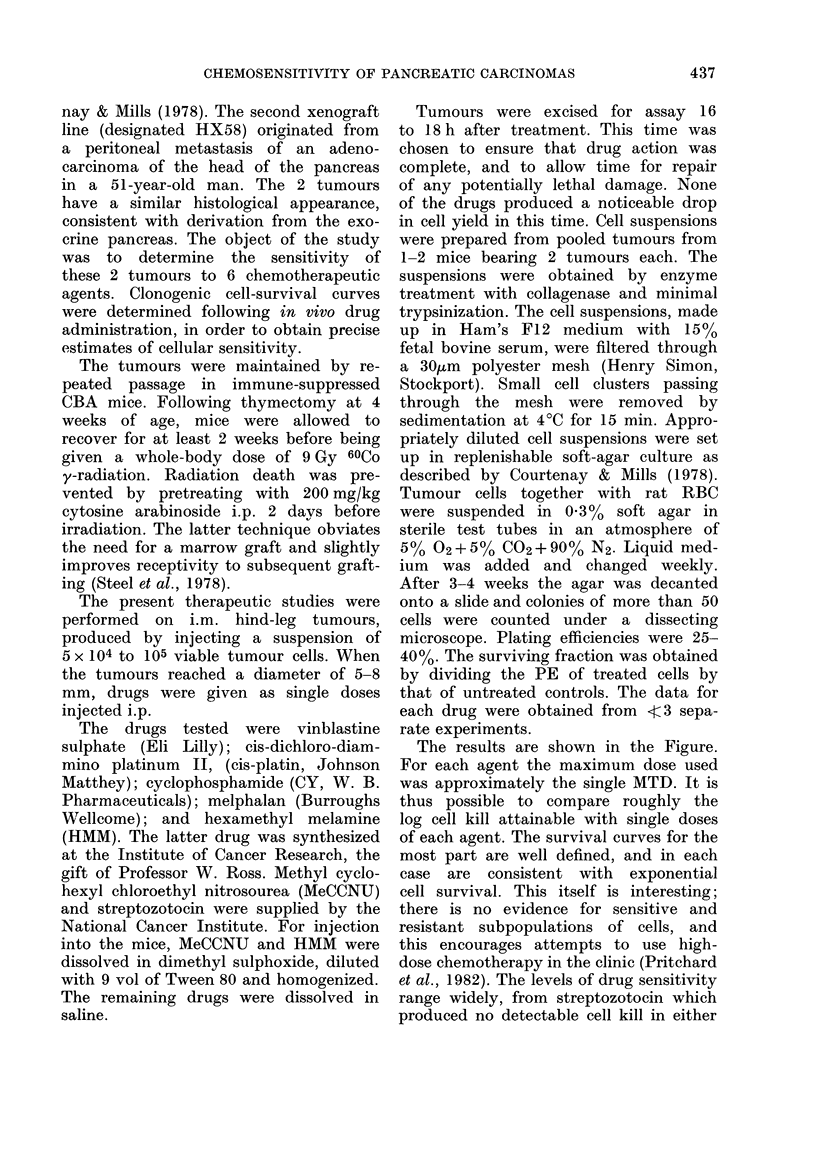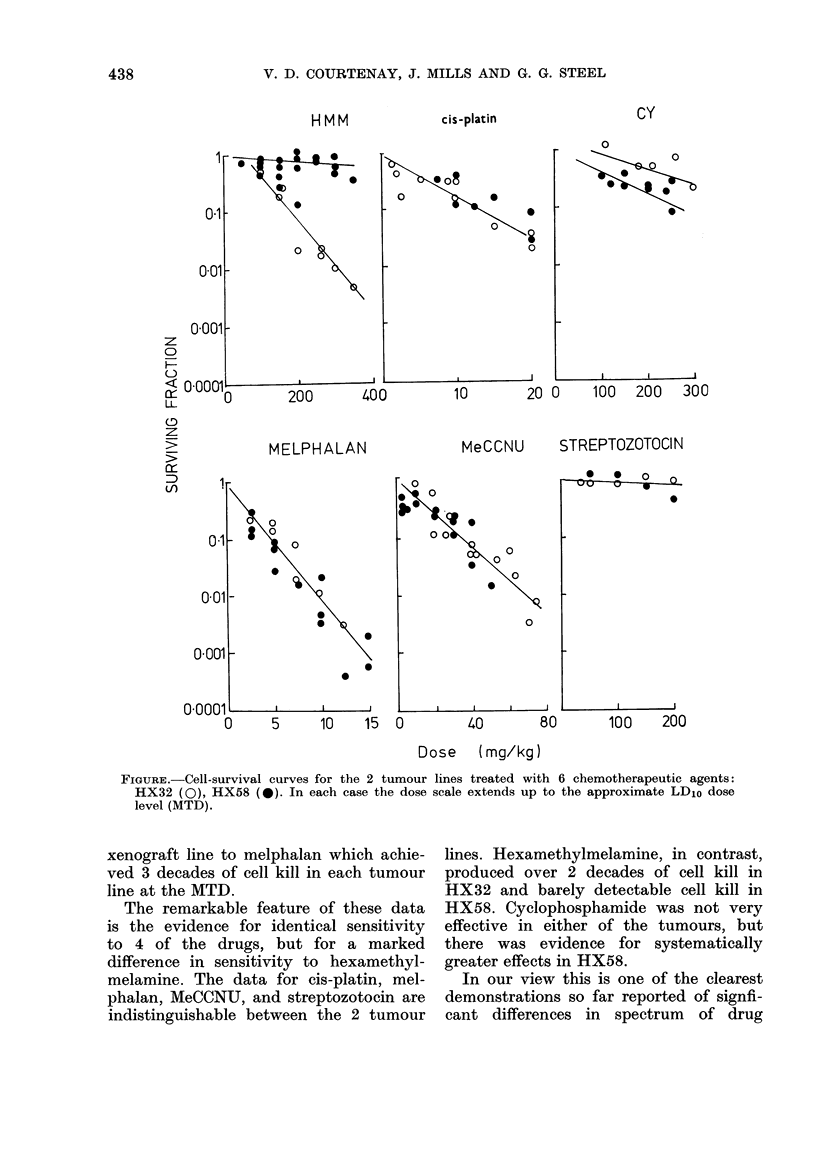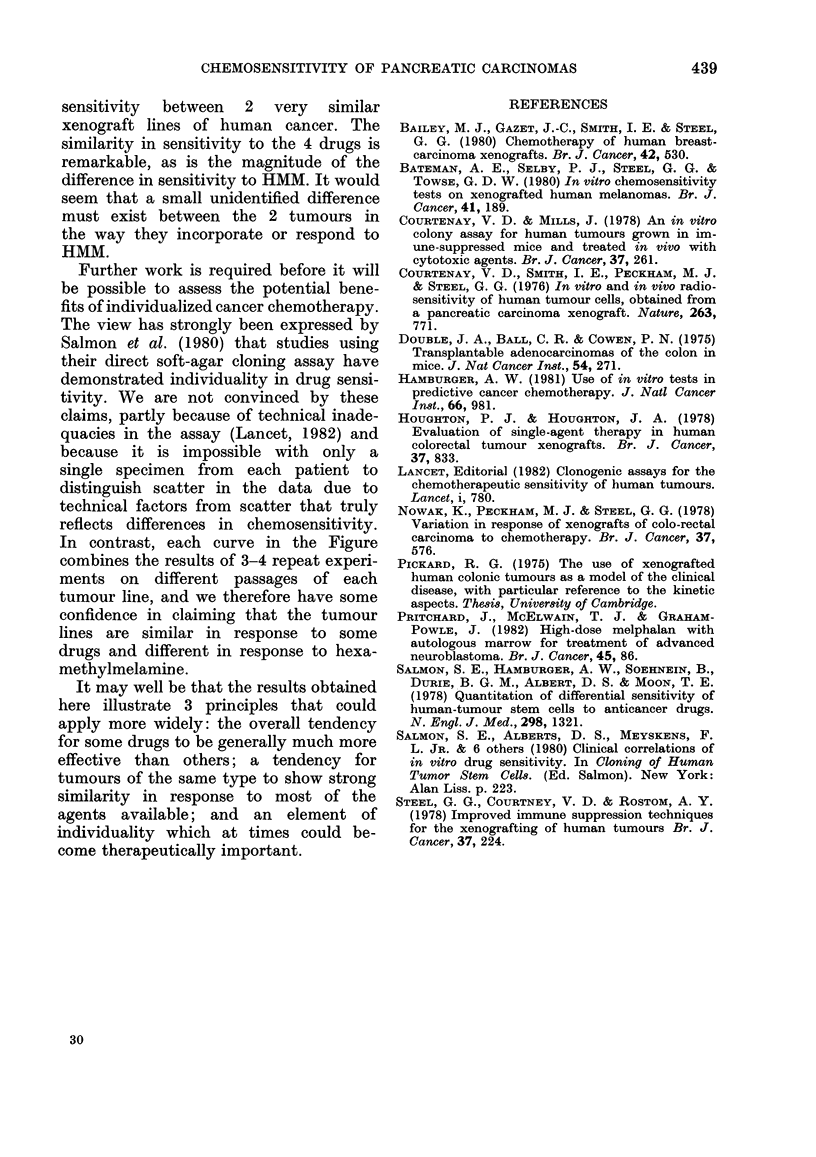# The spectrum of chemosensitivity of two human pancreatic carcinoma xenografts.

**DOI:** 10.1038/bjc.1982.221

**Published:** 1982-09

**Authors:** V. D. Courtenay, J. Mills, G. G. Steel


Br. J. Cancer (1982) 46, 436

### Short Communication

### THE SPECTRUM OF CHEMOSENSITIVITY OF TWO HUMAN PANCREATIC CARCINOMA XENOGRAFTS

V. D. COURTENAY, J. MILLS AND G. G. STEEL

*From the Radiotherapy Research Unit, Institute of Cancer Research, Belmont, Sutton, Surrey*

Received 3 March 1982 Accepted 14 May 1982

THERE IS growing interest in the individuality of human tumours with respect to cytotoxic drug sensitivity. That human tumours differ in response to chemotherapy is well known; some diseases, including choriocarcinoma and non-seminomatous tumours of the testis, usually respond well, in contrast, for example, to adenocarcinomas and squamous carcinomas in various sites, that respond poorly if at all. The question is, within a well-defined category of tumour, how wide is the range of drug sensitivity and is there firm evidence that the best drug to use may differ from one tumour to the next? In other words, among what is thought to be a group of otherwise identical tumours are there differences in the *spectrum* of drug response? There is widespread belief that such differences do exist. The current wave of interest in techniques by which the chemosensitivity of individual tumours can be evaluated (Salmon *et al.*, 1978; Hamburger, 1981) arises from the belief that individualizing chemotherapy can achieve useful improvements in treatment. Studies on a group of mouse colon tumours have also given evidence for their chemotherapeutic individuality (Double *et al.*, 1975).

Our programme of work on the chemotherapeutic response of human tumours grown as xenografts in immune-deprived mice has given some evidence in support of the individuality hypothesis. Nowak *et al.* (1978) examined the response to chemotherapy of 10 lines of colo-rectal carcinoma, evaluated by the growth-delay endpoint. Response to the 8 drugs that were used was generally poor, but there was some evidence that the drugs (melphalan, hexamethylmelamine and 5-fluorouracil) that on average did best, were not the best for every tumour. Houghton & Houghton (1978) came to a similar conclusion, also on colo-rectal cancer. In a study of breast-cancer xenografts, Bailey *et al.* (1980) examined the response of 5 tumour lines to 6 single agents and 2 drug combinations. Melphalan alone, and the 2 combinations, were on average the best treatments, but among these the ranking varied from one tumour line to the next. Bateman *et al.* (1980) used *in vitro* drug sensitivity tests to rank the response of 5 malignant melanoma xenografts to each of 8 chemotherapeutic agents. Here again there was a similar picture, with an overall trend in drug ranking (in this case favouring melphalan, MeCCNU and cis-platin) superimposed on which was some evidence for individuality. In none of these studies was the evidence for individuality statistically significant, but it encouraged further attempts to confirm this phenomenon.

The present work comprised a detailed study on two pancreatic carcinoma xenograft lines. The first, which we have called HX32, was established by Pickard (1975). It was used in the radiobiological study described by Courtenay *et al.* (1976) and is described in more detail by Courtenay & Mills (1978). The second xenograft line (designated HX58) originated from a peritoneal metastasis of an adenocarcinoma of the head of the pancreas in a 51-year-old man. The 2 tumours have a similar histological appearance, consistent with derivation from the exocrine pancreas. The object of the study was to determine the sensitivity of these 2 tumours to 6 chemotherapeutic agents. Clonogenic cell-survival curves were determined following *in vivo* drug administration, in order to obtain precise estimates of cellular sensitivity.

The tumours were maintained by repeated passage in immune-suppressed CBA mice. Following thymectomy at 4 weeks of age, mice were allowed to recover for at least 2 weeks before being given a whole-body dose of 9 Gy $^{60}$Co $\gamma$-radiation. Radiation death was prevented by pretreating with 200 mg/kg cytosine arabinoside i.p. 2 days before irradiation. The latter technique obviates the need for a marrow graft and slightly improves receptivity to subsequent grafting (Steel *et al.*, 1978).

The present therapeutic studies were performed on i.m. hind-leg tumours, produced by injecting a suspension of $5 \times 10^4$ to $10^5$ viable tumour cells. When the tumours reached a diameter of 5–8 mm, drugs were given as single doses injected i.p.

The drugs tested were vinblastine sulphate (Eli Lilly); cis-dichloro-diammino platinum II, (cis-platin, Johnson Matthey); cyclophosphamide (CY, W. B. Pharmaceuticals); melphalan (Burroughs Wellcome); and hexamethyl melamine (HMM). The latter drug was synthesized at the Institute of Cancer Research, the gift of Professor W. Ross. Methyl cyclohexyl chloroethyl nitrosourea (MeCCNU) and streptozotocin were supplied by the National Cancer Institute. For injection into the mice, MeCCNU and HMM were dissolved in dimethyl sulphoxide, diluted with 9 vol of Tween 80 and homogenized. The remaining drugs were dissolved in saline.

Tumours were excised for assay 16 to 18 h after treatment. This time was chosen to ensure that drug action was complete, and to allow time for repair of any potentially lethal damage. None of the drugs produced a noticeable drop in cell yield in this time. Cell suspensions were prepared from pooled tumours from 1–2 mice bearing 2 tumours each. The suspensions were obtained by enzyme treatment with collagenase and minimal trypsinization. The cell suspensions, made up in Ham's F12 medium with 15% fetal bovine serum, were filtered through a 30$\mu$m polyester mesh (Henry Simon, Stockport). Small cell clusters passing through the mesh were removed by sedimentation at 4°C for 15 min. Appropriately diluted cell suspensions were set up in replenishable soft-agar culture as described by Courtenay & Mills (1978). Tumour cells together with rat RBC were suspended in 0·3% soft agar in sterile test tubes in an atmosphere of 5% $O_2$ + 5% $CO_2$ + 90% $N_2$. Liquid medium was added and changed weekly. After 3–4 weeks the agar was decanted onto a slide and colonies of more than 50 cells were counted under a dissecting microscope. Plating efficiencies were 25–40%. The surviving fraction was obtained by dividing the PE of treated cells by that of untreated controls. The data for each drug were obtained from $\nless 3$ separate experiments.

The results are shown in the Figure. For each agent the maximum dose used was approximately the single MTD. It is thus possible to compare roughly the log cell kill attainable with single doses of each agent. The survival curves for the most part are well defined, and in each case are consistent with exponential cell survival. This itself is interesting; there is no evidence for sensitive and resistant subpopulations of cells, and this encourages attempts to use high-dose chemotherapy in the clinic (Pritchard *et al.*, 1982). The levels of drug sensitivity range widely, from streptozotocin which produced no detectable cell kill in either

FIGURE.—Cell-survival curves for the 2 tumour lines treated with 6 chemotherapeutic agents: HX32 (○), HX58 (●). In each case the dose scale extends up to the approximate $LD_{10}$ dose level (MTD).

xenograft line to melphalan which achieved 3 decades of cell kill in each tumour line at the MTD.

The remarkable feature of these data is the evidence for identical sensitivity to 4 of the drugs, but for a marked difference in sensitivity to hexamethylmelamine. The data for cis-platin, melphalan, MeCCNU, and streptozotocin are indistinguishable between the 2 tumour lines. Hexamethylmelamine, in contrast, produced over 2 decades of cell kill in HX32 and barely detectable cell kill in HX58. Cyclophosphamide was not very effective in either of the tumours, but there was evidence for systematically greater effects in HX58.

In our view this is one of the clearest demonstrations so far reported of signficant differences in spectrum of drug sensitivity between 2 very similar xenograft lines of human cancer. The similarity in sensitivity to the 4 drugs is remarkable, as is the magnitude of the difference in sensitivity to HMM. It would seem that a small unidentified difference must exist between the 2 tumours in the way they incorporate or respond to HMM.

Further work is required before it will be possible to assess the potential benefits of individualized cancer chemotherapy. The view has strongly been expressed by Salmon *et al.* (1980) that studies using their direct soft-agar cloning assay have demonstrated individuality in drug sensitivity. We are not convinced by these claims, partly because of technical inadequacies in the assay (Lancet, 1982) and because it is impossible with only a single specimen from each patient to distinguish scatter in the data due to technical factors from scatter that truly reflects differences in chemosensitivity. In contrast, each curve in the Figure combines the results of 3–4 repeat experiments on different passages of each tumour line, and we therefore have some confidence in claiming that the tumour lines are similar in response to some drugs and different in response to hexamethylmelamine.

It may well be that the results obtained here illustrate 3 principles that could apply more widely: the overall tendency for some drugs to be generally much more effective than others; a tendency for tumours of the same type to show strong similarity in response to most of the agents available; and an element of individuality which at times could become therapeutically important.

### REFERENCES

Bailey, M. J., Gazet, J.-C., Smith, I. E. & Steel, G. G. (1980) Chemotherapy of human breast-carcinoma xenografts. *Br. J. Cancer*, **42**, 530.

Bateman, A. E., Selby, P. J., Steel, G. G. & Towse, G. D. W. (1980) *In vitro* chemosensitivity tests on xenografted human melanomas. *Br. J. Cancer*, **41**, 189.

Courtenay, V. D. & Mills, J. (1978) An *in vitro* colony assay for human tumours grown in immune-suppressed mice and treated *in vivo* with cytotoxic agents. *Br. J. Cancer*, **37**, 261.

Courtenay, V. D., Smith, I. E., Peckham, M. J. & Steel, G. G. (1976) *In vitro* and *in vivo* radiosensitivity of human tumour cells, obtained from a pancreatic carcinoma xenograft. *Nature*, **263**, 771.

Double, J. A., Ball, C. R. & Cowen, P. N. (1975) Transplantable adenocarcinomas of the colon in mice. *J. Nat Cancer Inst.*, **54**, 271.

Hamburger, A. W. (1981) Use of *in vitro* tests in predictive cancer chemotherapy. *J. Natl Cancer Inst.*, **66**, 981.

Houghton, P. J. & Houghton, J. A. (1978) Evaluation of single-agent therapy in human colorectal tumour xenografts. *Br. J. Cancer*, **37**, 833.

Lancet, Editorial (1982) Clonogenic assays for the chemotherapeutic sensitivity of human tumours. *Lancet*, i, 780.

Nowak, K., Peckham, M. J. & Steel, G. G. (1978) Variation in response of xenografts of colo-rectal carcinoma to chemotherapy. *Br. J. Cancer*, **37**, 576.

Pickard, R. G. (1975) The use of xenografted human colonic tumours as a model of the clinical disease, with particular reference to the kinetic aspects. *Thesis, University of Cambridge*.

Pritchard, J., McElwain, T. J. & Graham-Powle, J. (1982) High-dose melphalan with autologous marrow for treatment of advanced neuroblastoma. *Br. J. Cancer*, **45**, 86.

Salmon, S. E., Hamburger, A. W., Soehnein, B., Durie, B. G. M., Albert, D. S. & Moon, T. E. (1978) Quantitation of differential sensitivity of human-tumour stem cells to anticancer drugs. *N. Engl. J. Med.*, **298**, 1321.

Salmon, S. E., Alberts, D. S., Meyskens, F. L. Jr. & 6 others (1980) Clinical correlations of *in vitro* drug sensitivity. In *Cloning of Human Tumor Stem Cells*. (Ed. Salmon). New York: Alan Liss. p. 223.

Steel, G. G., Courtney, V. D. & Rostom, A. Y. (1978) Improved immune suppression techniques for the xenografting of human tumours *Br. J. Cancer*, **37**, 224.